# Natural History of Aerosol Exposure with Marburg Virus in Rhesus Macaques

**DOI:** 10.3390/v8040087

**Published:** 2016-03-30

**Authors:** Evan C. Ewers, William D. Pratt, Nancy A. Twenhafel, Joshua Shamblin, Ginger Donnelly, Heather Esham, Carly Wlazlowski, Joshua C. Johnson, Miriam Botto, Lisa E. Hensley, Arthur J. Goff

**Affiliations:** 1 Department of Medicine, Tripler Army Medical Center, Honolulu, HI 96859, USA; evan.c.ewers.mil@mail.mil (E.C.E); nancy.a.twenhafel.mil@mail.mil (N.A.T.); arthur.j.goff.civ@mail.mil (A.J.G.); 2 US Army Medical Research Institute of Infectious Diseases, Fort Detrick, Frederick, MD 21702, USA; joshua.d.shamblin1.civ@mail.mil (J.S.); ginger.c.donnelly.ctr@mail.mil (G.D.); heather.l.esham.civ@mail.mil (H.E.); carly.b.wlazlowski.ctr@mail.mil (C.W.); miriam.a.botto.civ@mail.mil (M.B.); 3 Integrated Research Facility, National Institute of Allergy and Infectious Diseases, National Institutes of Health, Frederick, MD 21702, USA; joshua.johnson@nih.gov (J.C.J.); lisa.hensley@nih.gov (L.E.H.)

**Keywords:** filovirus, nonhuman primate, Marburg virus, aerosol, telemetry, animal model

## Abstract

Marburg virus causes severe and often lethal viral disease in humans, and there are currently no Food and Drug Administration (FDA) approved medical countermeasures. The sporadic occurrence of Marburg outbreaks does not allow for evaluation of countermeasures in humans, so therapeutic and vaccine candidates can only be approved through the FDA animal rule—a mechanism requiring well-characterized animal models in which efficacy would be evaluated. Here, we describe a natural history study where rhesus macaques were surgically implanted with telemetry devices and central venous catheters prior to aerosol exposure with Marburg-Angola virus, enabling continuous physiologic monitoring and blood sampling without anesthesia. After a three to four day incubation period, all animals developed fever, viremia, and lymphopenia before developing tachycardia, tachypnea, elevated liver enzymes, decreased liver function, azotemia, elevated D-dimer levels and elevated pro-inflammatory cytokines suggesting a systemic inflammatory response with organ failure. The final, terminal period began with the onset of sustained hypotension, dehydration progressed with signs of major organ hypoperfusion (hyperlactatemia, acute kidney injury, hypothermia), and ended with euthanasia or death. The most significant pathologic findings were marked infection of the respiratory lymphoid tissue with destruction of the tracheobronchial and mediastinal lymph nodes, and severe diffuse infection in the liver, and splenitis.

## 1. Introduction

Marburg virus (MARV) is a well-recognized cause of viral hemorrhagic fever outbreaks in humans, and together with multiple species of Ebolaviruses, and the newly discovered “Cuevaviruses”, make up the family *Filoviridae*. Currently, there are five recognized species of Ebolavirus—Zaire, Sudan, Bundibugyo, Reston, and Tai Forest species—and only one recognized species of MARV—*Marburg marburgvirus*—although several genetically distinct isolates have been associated with human outbreaks (Ci67, Popp, Ravn). Among the most notable of these is the Angola strain (MARV-Ang), which caused a devastating outbreak between 2004 and 2005.

The first recognized outbreak of Marburg virus disease was reported in 1967 among laboratory workers from Germany and Yugoslavia who had come into contact with infected nonhuman primate (NHP) tissues [1,2,3]. This initial outbreak had a reported case-fatality rate of 22%, with seven of 31 infected patients perishing. Although sporadic cases and small-scale outbreaks occurred in sub-Saharan Africa during the 1970s and 1980s, the outbreaks of MARV in the Democratic Republic of the Congo between 1998 and 2000, as well as the 2004 outbreak in Angola, showed that MARV has the capacity for wide-spread transmission and marked lethality [4,5,6,7,8,9]. Of note, the Angola outbreak was among the most deadly of all filovirus disease outbreaks with a reported case-fatality over 90% [8]. More recently, the 2014 West-African outbreak of Ebola virus disease, and subsequent cases particularly in the United States, demonstrates the capacity for outbreaks to occur even in non-endemic areas as a result of modern global travel [10]. In addition, a Soviet defector has claimed the USSR had attempted to develop aerosolized MARV and Ebolavirus as biological weapons [11].

Due to the remote locations of outbreaks in resource-limited environments, the most detailed clinical descriptions of MARV infection in humans comes from the original case reports from the 1967 outbreak, as well as several isolated cases and the epidemiologic studies of recent outbreaks [1,2,4,5,6,8,9,12]. Human infection with MARV occurs from direct contact with contaminated blood or bodily fluids, either from an infected person or animal [1,2,12,13]. The incubation period in humans ranges from two to 21 days, with an average onset of symptoms between nine and 16 days following infection. Patients typically develop generalized symptoms, such as fever, headache, malaise, and mylagias. Over the next three to five days, symptoms progress to include nausea, vomiting, diarrhea, abdominal pain, and a nonspecific macular, or maculopapular rash. Prostration, dyspnea, and generalized edema soon develop; coupled with disseminated intravascular coagulation (DIC), distributive shock, and multiple organ failure [2,12,14]. Death typically follows within eight to 16 days following symptom onset. Based on the available clinical data, it appears overt hemorrhage is not a hallmark of MARV infection, and the most common manifestations include hematemesis, hematochezia, melena, and mucosal bleeding. However, the presence of hemorrhagic manifestations was shown as a predictor of a fatality during the Congolese outbreak of 1998–2000 [9]. Human pathologic data is limited, but severe hepatocellular necrosis, lymphocytic depletion of the spleen and lymphatic tissues with evidence of hemorrhage and congestion, tubular necrosis of the kidneys, adrenal gland necrosis, and pancreatitis have been documented [2,4].

The majority of the data on pathogenesis, clinical disease, and pathology of MARV and Ebola virus infection comes from detailed experiments conducted on NHP models. As evidenced by these studies, filovirus infection can occur following introduction via intramuscular, conjunctival, oral, or aerosol routes [15,16,17,18,19]. Although considered largely pantropic viruses, dendritic cells, monocytes, macrophages, and Kupffer cells are initial targets of infection and origins of filoviral replication [20]. From there, the virus quickly infects hepatocytes, fibroblasts, fibroblastic reticular cells, and eventually endothelial cells. Initial infection of macrophages leads to increased pro-inflammatory cytokines including MIP-1α, MCP-1, IL-6, IL-18, and IL-1β. Vasoactive molecules such as nitric oxide, and TNF-related apoptosis-inducing ligand (TRAIL) have been detected [16,21,22,23], which have been associated with a number of hallmarks in the pathogenesis of filoviral hemorrhagic fevers. Lymphocyte apoptosis seen in Ebola virus-infected NHPs and humans is thought to occur through the hypersecretion of numerous cytokines and chemokines and up-regulation of TRAIL and Fas, leading to lymphopenia, blunted innate immune response, and a significant delay in adaptive immunity [20,22,24,25,26]. Additionally, bleeding diatheses observed in NHP and human infection results from DIC, which occurs through activation of tissue factor (TF)/Factor VII pathway, depletion of protein C levels, and endothelial damage [16,27,28,29]. Altogether, the experiments conducted on filovirus pathogenesis revealed that clinical manifestations are a result of both direct virus-mediated effect and subsequent massive pro-inflammatory cytokine release [18,20,21,22].

Currently, there are no approved therapies for humans infected with MARV, and due to the remote location and sporadic nature of outbreaks, clinical testing has been logistically and ethically difficult. Therefore, future therapies will likely need to seek approval through the US Food and Drug Administration’s “Animal Rule”, which allows animal models to be used for licensure data provided they accurately mimic human disease. To license such products against the potential biothreat would require the model to use the aerosol route of MARV exposure. To date, multiple animal models have been utilized for the study of filoviral infection including mice, hamsters, guinea pigs, and NHPs—baboons, African green monkeys, marmosets, and rhesus and cynomolgus macaques. Of these, macaques appear to most closely resemble human disease, making them ideal for the study of cellular, tissue, and systemic pathogenesis, and therapeutic modalities. While the pathology and pathogenesis are well-described, there is a relative paucity of physiologic and clinical data to further describe the disease course in these models, which would allow for the understanding of the pathophysiology occurring during the clinical course of MARV infection. Over the past decade, advances in radiotelemetry devices have allowed for the recording of physiologic measurements during the course of infection in animal models, including heart rate (HR), blood pressure (BP), temperature, and respiratory rate. Two recent studies have incorporated some of these physiologic parameters into evaluation of Ebola virus and MARV-Ang infection of NHPs [17,23]. However, neither of these studies evaluated MARV-Ang infection in rhesus macaques as a model of human disease. Here, we describe the natural history of MARV infection in rhesus macaques infected through an aerosol route, providing detailed insight into the clinical and pathophysiologic components of the disease process, and demonstrate the applicability of rhesus macaques as a model of MARV-Ang infection.

## 2. Materials and Methods

### 2.1. Ethics Statement and Animal Care and Use

This research study was approved by the U.S. Army Medical Research Institute of Infectious Diseases (USAMRIID) Institutional Animal Care and Use Committee (IACUC). The specific national regulations and guidelines to which this animal care and use protocol adheres are the following: (1) 7 United States Code, Sections 2131–2159, Chapter 54 “Animal Welfare Act”, and Code of Federal Regulations, Chapter 1, Subchapter A, Parts 1–4 “Animal Welfare Regulations”; (2) Health Research Extension Act of 1985, Public Law 99-158 “Animals in Research” and the Public Health Service Policy in Humane Care and Use of Laboratory Animals; (3) Biosafety in Microbiological and Biomedical Laboratories, 5^th^ Edition, National Institute of Health, Human and Health Services Publication (CDC) 21-112; (4) Army Regulation 40-33 “The Care and Use of Animals in DOD Research, Development, Test and Evaluation or Training Programs” and (5) DOD Instruction 3216.01 “Use of Animals in DOD Programs”. USAMRIID is accredited by AAALAC/I, and uses the publication titled “The Guide for the Care and Use of Laboratory Animals”, 8th Edition, Institute for Laboratory Animal Research, National Research Council, as a guideline for evaluation and accreditation of program and it is based on the national regulations and guidelines for animal care and use programs. Animals were housed in individual metal cages meeting current standards after the surgical procedures, and for the duration of the housing period in biosafety level 4. Animals were pair-housed as much as possible prior to the surgical procedures. Animals were acclimated to wear the Lomir jackets for 3–7 days prior to the central venous catheter implantation. Room environment was centrally controlled by an HVAC system that maintains room humidity between 30% and 70% and temperature between 22.2 and 23.3 °C. The room ventilation was 10–15 air changes per hour. Animals were provided pelleted commercially produced monkey diet twice daily prior to moving them to biocontainment where they were fed once daily. Potable water was provided *ad libitum* from an automatic watering system. In addition, animals were provided manipulanda (toys, metal mirrors), foraging devices, treats and fruits as environmental enrichment. Analgesia was not used during the study and scientific justification for this was approved by the IACUC. Animals were evaluated at least twice daily beginning 3 days prior to exposure for appetite, cough, nasal-ocular discharge, rash, hemorrhagic manifestations, neurological signs, urine and stool output, as well as lethargy. Following the development of clinical signs, animals were checked multiple times daily by trained, IACUC-approved staff using an IACUC-approved euthanasia scoring system. When clinical observations and scores of animals reached defined levels based on the approved IACUC protocol, animals were euthanized with a pentobarbital-based euthanasia solution under anesthesia to minimize pain and distress.

### 2.2. Non-Human Primates and Implantation of Devices

Five healthy (3 male, 2 female) rhesus macaques (*Macaca mulatta*) weighing between 5.7 and 7.2 kg were obtained from commercial vendors and evaluated for normal health and behavior prior to starting the study. All five macaques were surgically implanted with T27F-1B radiotelemetry devices (Konigsberg Instruments, Pasadena, CA, USA) five months prior to the start of the experiment, and were allowed to fully recover. Central venous catheters (CVCs) were surgically inserted in the internal jugular vein of the test animals 7 days prior to the start of experiments, and they were fitted with non-restraining jackets. The catheters were attached to a tether system following implantation, and a heparin-saline (10 units/mL) lock solution was used to maintain catheter patency. Surgeries were done under gas anesthesia and, if necessary, post-operational buprenorphine analgesia to minimize pain and distress.

### 2.3. Virus Strain and Aerosol Exposure

The MARV-Ang strain (GenBank accession number DQ447654.1) used in this aerosol challenge was isolated from a pediatric patient in 2005 and was passaged twice in Vero cells prior to production of seed stock [30]. The stock used in this experiment was analyzed for purity, sterility, hematological parameters, nucleotide sequence, morphology and confirmation of virulence.

Prior to MARV challenge, a sham aerosol spray using virus was performed in order to calculate the spray factor, which was used to determine the time necessary to achieve the target dose of 1000 pfu. The 5 subjects were exposed to MARV-Ang in a Head Only Automated Bioaerosol Exposure System housed in a Class III biological safety cabinet in a BSL-4 suite. Animals were anesthetized with tiletamine/zolazepam (3 mg/kg), prior to placement in the exposure chamber, and whole-body plethysmography was used to calculate the aerosol challenge dose. Samples of pre-spray suspension and the aerosol were collected from the exposure chamber for each subject, and utilized in plaque assay to determine each subject’s inhaled dose (Table 1). The start of Day 0 was defined as the time the animal was returned to its cage after exposure.

### 2.4. Telemetry Monitoring

The implanted radiotelemetry devices allowed for the continuous monitoring of body temperature, aortic pressure (AOP), left ventricular pressure (LVP), heart rate (HR), and respiratory rate (RR) throughout the baseline and study periods. The telemetry data were captured and analyzed using the Notocord-hem Evolution software platform (Version 4.3.0.20, Notocord Inc., Newark, NJ, USA). Thirty-minute averages of the above listed physiologic parameters were calculated for each subject. Telemetry data obtained during the four days prior to MARV challenge were used to calculate baseline values, and provided the average and standard deviation (SD) of each 30-min time period of a 24-h day. Using the average and SD determinations from the baseline physiologic data, values +/− 3 SD of the corresponding baseline value were considered significant. For temperature, this was used to calculate daily fever duration (h), daily percent fever (% of 24-h period with significant temperature elevations), and fever-hours (°C-h; the sum of significant temperature elevations). Additionally, temperature data obtained were also classified according to traditional clinical classifications. Fever was defined as >1.5 °C above corresponding baseline value, hyperpyrexia was defined as >3.0 °C above, and hypothermia was defined as >2.0 °C below their corresponding baseline values.

### 2.5. Clinical Observations and Euthanasia Criteria

Macaques were evaluated at least twice daily beginning 3 days prior to exposure for appetite, cough, nasal-ocular discharge, rash, hemorrhagic manifestations, neurological signs, urine and stool output, as well as lethargy. Animals were evaluated using a euthanasia scoring system, which measured responsiveness, seizure activity, recumbancy, and respiratory difficulty. Macaques with overall scores of 8 or higher, or macaques that were unresponsive to painful stimuli were humanely euthanized according to standard protocol.

### 2.6. Clinical Pathology

Blood was sampled daily beginning three days prior to virus exposure to measure hematology, serum chemistry, liver enzymes, coagulation parameters, fibrin degradation products, viremia, and the cytokine response. Blood was drawn through the CVC or through the saphenous vein if the CVC failed. If blood was drawn through the saphenous vein, macaque was anesthetized with ketamine (10 mg/kg). The CVC was flushed and locked with heparinized saline following blood draws, as above.

A complete blood count (CBC) was collected for hematologic data using the HemaVet 950FS Hematology Analyzer (Drew Scientific, Inc., Dallas, TX, USA), and 13 clinical chemistry parameters were obtained using a Piccolo Analyzer (Abaxis, Union City, CA, USA) within 4 h of the initial blood draw. Blood gases, pH, and lactate levels, as well as coagulation parameters, were assessed with an iSTAT portable clinical analyzer (Abbott Laboratories, Princeton, NJ, USA) by placing blood onto iSTAT CG4+ and PT/INR cartridges, respectively. D-dimer levels were measured using an Asserachrom D-Dimer Enzyme Immunoassay kit (Diagnostica Stago Inc., Parsippany, NJ, USA) according to the manufacturer’s instructions. Cytokine levels in plasma were determined using a 23-plex NHP cytokine panel acquired with a Bio-Plex 100 xMAP instrument (Bio-Rad Life Science Group, Hercules, CA, USA) according to the manufacturer’s instructions.

### 2.7. Measurement of Viremia

Two methods were employed to detect viremia in the exposed macaques. First, we used a standard plaque assay on Vero E6 cells for quantification of the viral stock: the aerosol exposure for each animal on Day 0, and EDTA plasma from each macaque, daily. One-step quantitative RT-PCR was also performed on serum samples obtained daily from subjects beginning on Day 0, and continuing until the animal was euthanized or succumbed to the disease. Reactions were performed in a LightCycler 480 (Roche, Indianapolis, IN, USA). Forward primer (CCAGTTCCAGCAATTACATACATACA), reverse primer (GCACCGTGGTCAGCATAAGGA) and TaqMan probe (CAATACCTTAACCCC C-MGBNGQ) allowed amplification of the MARV glycoprotein gene. Cycling conditions used were reverse transcription at 50 °C for 15 min, initial denaturation at 95 °C for 5 min; followed by 45 cycles of denaturation at 95 °C for 1 s, and annealing, synthesis and single acquisition at 60 °C for 20 s; and final cooling at 40 °C for 30 s. Quantification was based on viral RNA standard using the LC480 software.

### 2.8. Histopathology and Immunohistochemistry

A veterinary pathologist performed full necropsies on all subjects under BSL-4 conditions following death or euthanasia. Tissues for histopathologic examination were fixed by immersion in 10% neutral buffered formalin for 21 days under BSL-4 conditions. Formalin-fixed tissues were removed from BSL-4, trimmed, processed, and then stained with hematoxylin and eosin. All histopathologic evaluation was conducted using a light microscope.

Immunohistochemistry was performed on select sections using an Envision-PO (Dako Inc., Carpinteria, CA, USA) kit with an anti-MARV mouse monoclonal antibody (#1286) at a 1:600 dilution, according to manufacturer’s recommendations. Sections were deparaffined and peroxidase blocked, then covered with primary antibody and incubated at room temperature for 30 min. They were then rinsed, and the secondary antibody was applied for 30 min. The sections were rinsed again, and a substrate-chromogen solution was applied for 5 min. The substrate was rinsed off, and hematoxylin was applied followed by rinsing, dehydration, and coverslipping. The presence of antigen was documented by + = minimal to mild antigen (1–100 positive cells); ++ = moderate (100–500 positive cells); or +++ = marked (500+ positive cells).

## 3. Results

### 3.1. Clinical Observations

In order to analyze the natural history of MARV-Ang infection in rhesus macaques, 5 healthy animals were exposed to a target dose of 1000 pfu of virus through an aerosol route (Table 1). The challenge dose was uniformly fatal in this experiment, with a mean time to death of 7.4 days. All subjects exhibited a remarkably similar clinical course with signs of illness beginning on post-infection day (PID) 6 and culminating in death or euthanasia on PID 7 or 8. Clinical signs were not seen until PID 6, when all subjects demonstrated decreased appetite and signs of depressed activity. On PID 7, all subjects were anorexic, 2 developed facial rash, and 1 displayed a rash on the arms and groin. Subject 2 progressed in its clinical course, became moribund, and was euthanized. On PID 8, Subjects 4 and 5 were found dead in the morning, and finally, Subjects 1 and 3 were euthanized after becoming recumbent and developing labored breathing. Overall, the most common observed manifestations of early systemic illness were decreased appetite (5/5), vomiting (4/5), and decreased responsiveness (4/5). Advanced illness was marked by anorexia (5/5), mild rash (3/5), decreased neurologic functioning (2/5) and tachypnea (3/5).

### 3.2. Viremia

Viremia was assessed using both a standard plaque assay from plasma and quantitative RT-PCR on subject serum (Table 1, Figure S1). Plaque assay counts revealed first detectable viremia on day PID 4, which continued to rise until peaking on PID 6 or 7. However, daily plaque counts are not available for all subjects due to limited plasma samples. The RT-PCR results closely adhered to plaque assay curves for all subjects. All 5 macaques had detectable levels of MARV-Ang by PID 4, correlating with the onset of fever (Figure S2), but preceding clinical observations of disease. Viral load in all macaques clustered tightly together, and peaked on PID 7, with levels around 10^8^ pfu/mL. For the 3 animals that survived to PID 8, their viral load plateaued (2/3) and decreased (1/3). Unsurprisingly, increased viral burden was observed with concomitant worsening clinical signs, elevated liver enzymes, decreased WBC count, and increase of pro-inflammatory mediators.

### 3.3. Telemetry Monitoring

**Figure 1:**
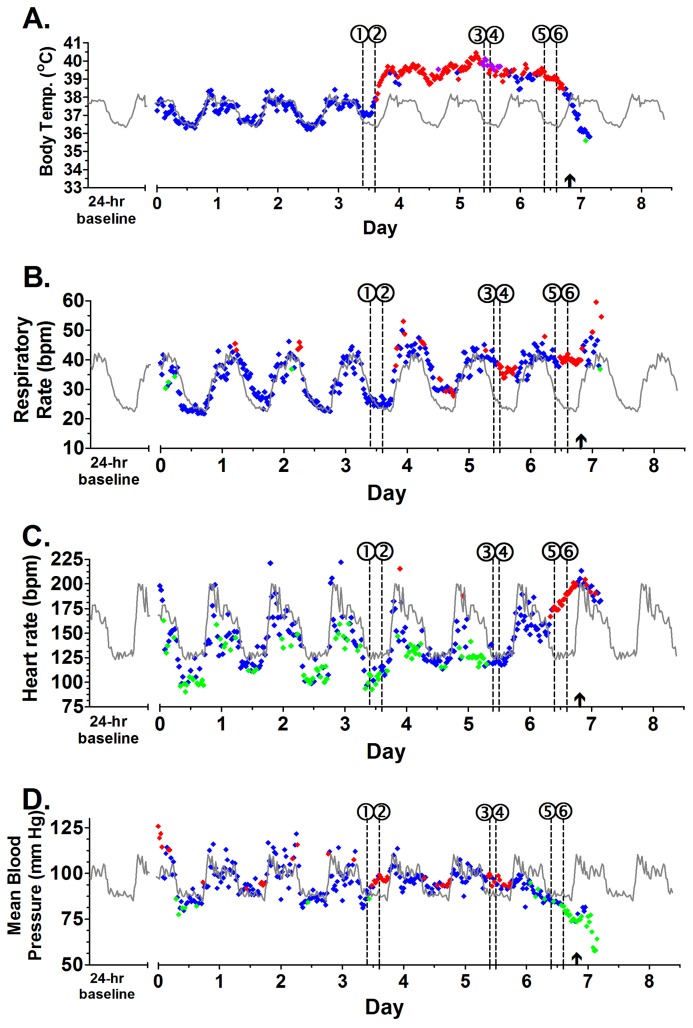
Running plot of body temperature, respiratory rate, heart rate, and blood pressure for Subject 2. (**A**) Body temperature values displaying normal (♦), fever (♦), hyperpyrexia (♦), and hypothermia (♦); (**B**) Respiratory rate; (**C**) Heart rate; (**D**) Blood pressure. Values + 3 SD (♦) or − 3 SD (♦) from baseline were considered statistically significant; Values <3 SD (♦) were not significant. Baseline values are seen in gray (**–**). Circled numbers indicate start of significant response for temperature (1), fever (2), hyperpyrexia (3), respiratory rate (4), heart rate (5), and blood pressure (6). A arrow (**↑**) marks the time when macaque was anesthetized with ketamine (10 mg/kg) for blood draw.

In order to understand the underlying pathophysiology behind the clinical presentation of Marburg hemorrhagic fever, all 5 subjects were implanted with T27F-1B implants, allowing for continuous monitoring of temperature, blood pressure, respiratory rate, and cardiac activity with minimal animal manipulation. All 5 macaques demonstrated normal diurnal variations in body temperature, heart rate, blood pressure, and respiratory rate with increases during the light hours and decreases during the dark hours prior to infection. Following aerosol exposure, all animals continued to demonstrate diurnal variation for the first 3–4 days. By the end of PID 3, two macaques developed sustained fever of 1.5 °C above the corresponding baseline values, and all 5 subjects were clinically and statistically febrile by PID 4 (Figure 1, Figures S2–S6). All subjects maintained a fever with periodic episodes of hyperpyrexia (>3 °C above corresponding baseline value) until the final 12–18 h of illness, when macaques exhibited loss of temperature regulation, and a profound decrease in temperature was observed. Three subjects had sustained periods of hypothermia (>1.5 °C below corresponding baseline value) in the final hours prior to death or euthanasia.

The other physiologic parameters—heart rate, respiratory rate, and blood pressure—showed deviations from baseline values following the onset of fever (Figure 1; Figures S3–S6). Typically, an increased respiratory rate was observed between 18 and 36 h following the onset of fever (4/5). For most subjects (4/5), the onset of hypotension was observed with concomitant sustained tachycardia, suggesting an initial state of compensated shock, followed shortly by a decompensation period. Only Subject 3 demonstrated bradycardia at the onset of its clinical course with only slight evidence of circulatory compensation at the onset of hypotension. In the final 3–6 h of illness prior to death or euthanasia, decreases in respiratory rate, heart rate, and continued hypotension were observed for all subjects. Based on the obtained telemetry data, MARV-Ang infection in rhesus macaques appears to have three distinct clinical stages: (1) an early clinical period that occurs from the onset of fever to sustained tachycardia or tachypnea; (2) a late clinical period lasting from the onset of sustained tachycardia or tachypnea to the onset of sustained hypotension; and (3) a terminal period beginning with the onset of profound hypotension to death or euthanasia.

### 3.4. Hematology

A Hemavet blood analyzer was used to assess changes in blood cell types following exposure to MARV-Ang (Figure S7). All macaques displayed stable WBC counts until PID 4, when overall counts decreased, reaching nadir on PID 6. On PID 7, there was a sharp increase and developing leukocytosis which was sustained in all subjects through euthanasia or death. Neutrophil counts displayed no discernible trends until PID 6, when a sharp increase in cell number was observed. Mononuclear cell (lymphocytes and monocytes) counts showed steady, dramatic decrease between day 0 and PID 6 (2.4–6.4 fold and 24 fold, respectively), which preceded very sharp increases (2.4–6.4-fold for lymphocytes, 1–6 fold for monocytes) on PID 7 and continued until death. Platelets showed little trend until PID 3, when counts decreased in all macaques (0.5–2-fold). However, profound thrombocytopenia was not observed, and sharp increases in platelet counts were observed beginning on day 7.

### 3.5. Clinical Chemistries, Blood Gases, and Coagulation Panel

**Figure 2:**
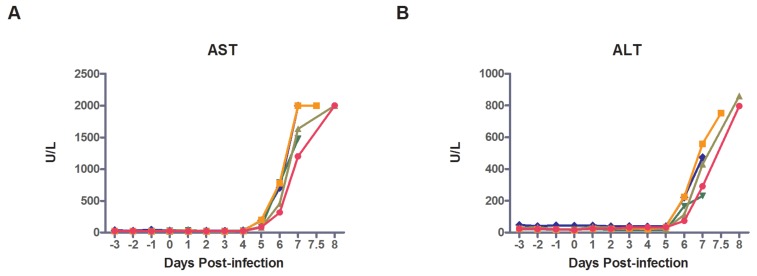

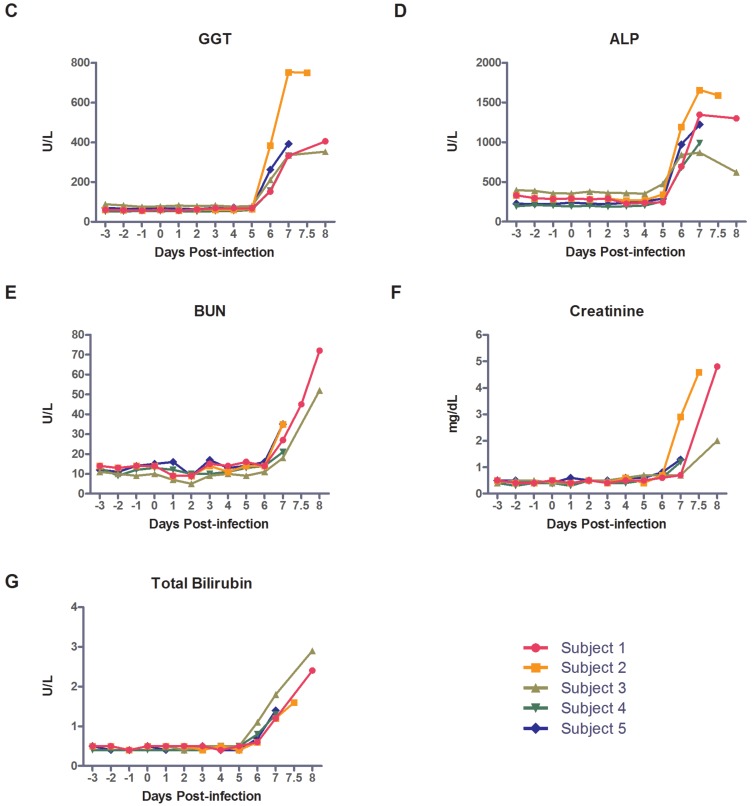
Hepatorenal function. Graphs demonstrate changes over time as determined by daily metabolic tests. The liver enzymes (**A**) AST; (**B**) ALT; (**C**) GGT; and (**D**) ALP were measured; (**E**) BUN; (**F**) Creatinine and (**G**) total bilirubin were measured to determine renal function.

Measurements of 13 different serum chemistry parameters were determined using a Piccolo analyzer beginning 3 days prior to MARV-Ang exposure. Chemical analytes showed no significant change from baseline values until PID 6, with the exception of steadily decreasing amylase levels beginning on day 4 (Figure S8). The most significant changes from baseline throughout the course of the clinical disease were observed increases in liver enzymes: ALT (10–36-fold); AST (54–83-fold); GGT (5.6–12.5-fold); and ALP (2.2–4.8-fold) (Figure 2). Peak AST levels of Subjects 1–3, were above the level of detection (>2000 U/L). Similar to other studies in filoviruses, an AST:ALT ratio >2:1 was observed [12,18,30]. Serum levels of albumin showed decreases from baseline on day PID 6 or 7, which coincided with increasing transaminases, and may be clinically indicative of decreasing liver function and/or capillary leakage from the acute inflammatory state (Figure S8). Terminal increases in BUN and creatinine were observed in all macaques, suggesting acute renal failure, a finding which has also been echoed in both human and NHP filovirus literature [18,23,31]. The decreases in serum calcium from baseline by PID 6/7 are likely due to the acute inflammatory response and the observed multisystem organ dysfunction seen in the infected macaques.

Analysis of blood gases using an iStat CG4+ cartridge revealed changes in pH, PCO_2_, and lactate, with first deviations from baseline noted on PID 7 (Figure S9). Increasing levels of lactate were associated with decrease in serum pH, consistent with a metabolic acidosis, which was most marked in Subject 2. A concomitant decrease in PCO_2_ accompanied increasing respiratory rate, indicative of the normal physiology compensatory mechanism to maintain acid-base homeostasis. Interestingly, other blood gas parameters including PO_2_, and HCO_3_ showed little or no deviation from baseline values (Figure S9).

**Figure 3:**
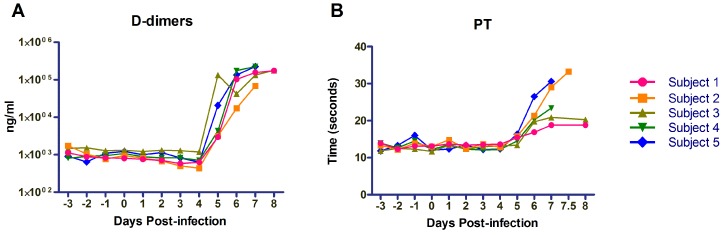
Coagulation parameters. (**A**) Pro-thrombin time was measured using an I-stat CG4+ cartridge starting on PID-3; (**B**) D-dimer levels were measured beginning on PID-3 and run in triplicate.

Since coagulopathy is a well-known hallmark of filoviral hemorrhagic fevers, a PT/INR coagulation cartridge was used to measure the functionality of the extrinsic coagulation pathway throughout the course of the disease. Additionally, D-dimer levels were also drawn to measure fibrin degradation as an indicator of DIC. The results in Figure 3 demonstrate the onset of coagulation abnormalities beginning on PID 6. Continued increases in D-dimer levels and PT times were seen in Subjects 2, 4, and 5 from Day 6 to death or euthanasia, while Subjects 1 and 3 PT values remain stable on PIDs 7 and 8. All macaques showed elevations in D-dimer levels from baseline values starting on PID 4 or 5, with elevations throughout disease course ranging between 50 and 200-fold over baseline values.

### 3.6. Analysis of Circulating Cytokines

Twenty-three different cytokines were measured daily throughout the experiment to determine the extent of anti- and pro-inflammatory mediators associated with MARV-Ang infection. Trends were not observed in circulating cytokine levels prior to the onset of clinical disease in all macaques. Of the cytokines that demonstrated moderate or substantial increases over baseline values, most began increasing by PID 5 or 6, and continued to rise through the peak of disease (Figure S10). Cytokines G-CSF, IFN-γ, IL-1ra, IL-1α, IL-1β, IL-5, IL-6, IL-18, MCP-1, MIP-1α, MIP-1β, TNF-α, displayed moderate to substantial increases from baseline, particularly noted during the terminal periods on PID 7. Levels of IL-13, IL-17, and VEGF increased slightly in the terminal period. A few cytokines were shown to have no distinguished trend or increases following infection: GM-CSF, IL-2, IL-12, and IL-4.

### 3.7. Histopathology and Immunohistochemistry

Necropsy findings demonstrated enlarged tracheobronchial lymph nodes (5/5), pale liver (5/5), friable spleens (2/5), and macular rash (3/5). Two subjects (1 and 2) had evidence of mucosal bleeding found on necropsy. The most profound histologic changes consistently present among subjects included lymphadenitis of tracheobronchial and mediastinal lymph nodes with nearly total loss of lymphocytes; severe degeneration and hepatocellular necrosis with myriad intracytoplasmic inclusion bodies (affecting nearly every cell in the liver); splenitis with profound loss of white pulp (lymphoid apoptosis); multisystemic vasculitis and perivasculitis; multisystem fibrin accumulation and vascular thromboemboli; and rarely hemorrhage. Lung pathology revealed evidence of vascular congestion (all subjects) and edema in Subjects 3–5 grossly. Two subjects (3 and 5) had lungs adherent to the parietal pleura, although it is unclear if these changes were due to implantation of the radiotelemetry devices. Histopathologic changes in the lung were notable for interstitial edema and fibrin deposition, and alveolar histiocytosis in all animals, although Subjects 1 and 2 demonstrated only mild changes, while Subjects 3–5 were noted to have moderate pathologic changes. Results of MARV-Ang IHC (Table S1) revealed moderate to strong positivity in liver, spleen, and tracheobronchial and mediastinal lymph nodes in all subjects. Overall, these findings are largely consistent with those published on cynomolgus macaques [17] and rhesus macaques [19] infected with MARV-Ang by the aerosol route.

## 4. Discussion

Despite the focus on the underlying molecular and cellular pathogenesis of filoviruses, and the intense focus on preventive and post-exposure prophylactic vaccines, there remains a paucity of clinical data to describe the pathophysiology of MARV-Ang infection. However, recent animal studies have incorporated telemetry devices for physiologic monitoring to better describe the disease pathogenesis [17,23]. Here, we sought to expand this knowledge by describing the natural progression of MARV-Ang infection in rhesus macaques.

**Figure 4:**
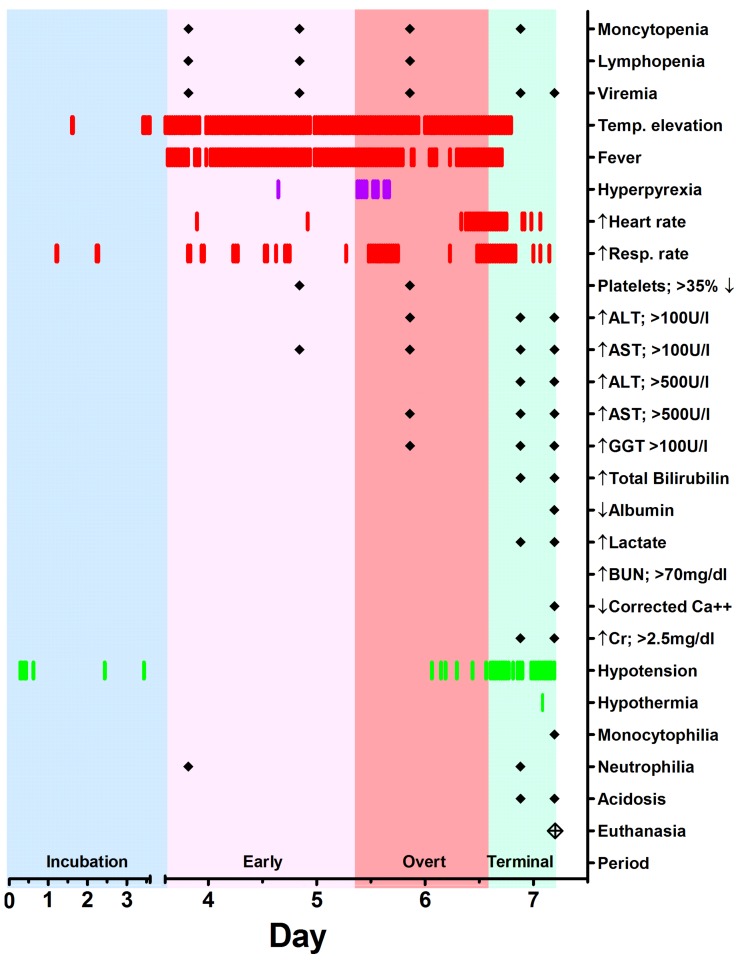
Example of summary of significant physiologic and laboratory findings, here from Subject 2. A red line (|) indicates significant elevation from baseline for physiologic data during the 30-min interval average as +3 SD from baseline values for heart rate and respiratory rate, or 1.5 °C (for fever only). A purple (|) indicates hyperpyrexia (>3 °C above baseline values). A green line (|) indicates +3 SD decline in blood pressure from baseline value for that 30-min interval average. Viremia is defined as presence of viral RNA in blood. AST = Aspartate aminotransferase. ALT = Alanine aminotransferase. GGT = Gamma glutamyltransferase. ↓ Albumin defined as 20% reduction in baseline values. ↑ Lactate defined as level about 5 mmol/L. BUN = blood urea nitrogen. ↓ Corrected Ca^2+^ defined as below 8.0 mg/dL. Cr = creatinine. Neutrophilia is defined as a 100% increase in baseline values. Lymphopenia is defined as 50% reduction below baseline values. Incubation period (blue) defined as time of challenge to onset of sustained fever response (>1.5 °C above baseline for 2-h). Early clinical period (light pink) defined as onset of sustained fever to onset of sustained tachycardia or tachypnea (+3 SD above baseline values). Overt clinical period (dark pink) defined as onset of sustained tachycardia or tachypnea until onset of sustained hypotension (+3 SD decline in blood pressure from baseline values). Terminal period (green) defined as onset of sustained hypotension until death or euthanasia.

Aerosol infection of rhesus macaques with MARV-Ang resulted in a uniformly lethal disease course with a mean time to death of 7.4 days, similar to that reported with parenteral infection [16]. The clinical course observed in this study was consistent with published clinical features of both human and NHP filoviral infections [2,9,12,14,16,17,18,23], which consisted of vomiting, fever, rash, lymphopenia, hepatitis, and severe coagulopathy clinically manifested as mucosal bleeding. Utilizing the physiologic and laboratory data, the course of MARV-Ang infection in rhesus macaques can be divided into four basic periods following exposure (Figure 4). Subjects experienced a short incubation period (three to four days) in which few signs were observed, although lymphocytopenia and monocytopenia were detected in all subjects. An early clinical phase began with the onset of sustained fever, and was accompanied with detectable virus via RT-PCR, and decreased platelets in 2/5 subjects. The disease course progressed to an overt clinical period marked by the onset of sustained tachycardia or tachypnea as determined by significant (±3 SD) changes from the animal’s baseline. Interestingly, while the incubation period for the virus was largely uniform between subjects, the duration of the early clinical period varied from only a few hours (Subjects 1 and 5), to several days. The overt clinical period was more pronounced, and consisted of physiologic, hematologic, and biochemical abnormalities in the macaques. These included tachypnea, tachycardia, lymphopenia, elevated liver enzymes, and elevated D-dimer levels and increased PT suggesting the onset of organ failure and coagulopathy. Altogether, the early and overt clinical periods last a total of two to three days once fever develops. The final, terminal period began with the onset of sustained hypotension, and included temperature decreases or hypothermia, as well as mucosal hemorrhage, macular rash, azotemia, renal insult, and metabolic acidosis in Subject 2. As determined by telemetry data, the stages of MARV-Ang infection in macaques mirror previously described stages (generalization, early organ, and late organ) in human disease based on the 1967 outbreak [12,32]. While the human disease course includes a convalescent stage with its own set of clinical characteristics, no such comparison could be made since this model was uniformly lethal. The gross and histopathological findings reported here compare well with those previously reported in rhesus macaques infected with MARV-Ang [17,20].

A similar physiologic course is observed in human cases of sepsis: a systemic inflammatory response with initially fever, tachycardia, hypotension, and tachypnea. Without adequate resuscitation, circulatory collapse ensues leading to hypotension, organ failure, terminal hypothermia, cardiopulmonary depression and death [33]. The clinical and telemetry results observed followed a similar progression. The initial febrile response coincided with the detection of virus in the serum, and was associated with blunting or obliteration of the normal diurnal variation observed in the pre-exposure time period, which was seen in previous studies for both Ebola and MARV-Ang infected macaques [17,23]. During the course of human filovirus disease, temperature has been noted to alternate between periods of fever and hypothermia, although the clinical course is marked by a fever until the infection advances and hypothermia develops in the terminal stages [2,4,12,14,31]. An increased heart rate usually accompanies fever in various clinical scenarios and has been reported in macaque infection with Ebola virus, as well as during the course of sepsis in humans [23,33]. In contrast, we found that Subjects 2–5 had delays in HR increases following the onset of fever by several days, and interestingly, the HR only increased with the onset of hypotension, indicating relative bradycardia during the course of MARV-Ang infection. In humans, this was reported during the 1967 MARV outbreak [32], as well as being associated with Dengue hemorrhagic fever, typhoid, and leptosporosis [34]. At present, the mechanism of relative bradycardia is unknown, but clinically this might serve as a distinguishing point between MARV and Ebola virus infections, although larger studies would be needed to determine such potential. Tachycardia was demonstrated, however, in all subjects during the hypotensive periods, as has been documented in both humans and macaques [2,14,23,31,35]. To varying degrees, subjects displayed adequate control of blood pressure until the final 18–36 h prior to death or euthanasia, when hypotension uniformly began to set in—an observation seen in Ebola virus infected macaques and humans; although, Kortepeter *et al.* showed longer periods (24–72 h) of hypotension prior to death in their control groups than we have in this present study [23]. Despite needing to use LV pressures as a proxy for systemic BP in Subject 5, there were significant decreases during the terminal period, consistent with decreased systemic intravascular pressures. Respiratory rate increase during the febrile and hypotensive periods was consistent with filovirus infection in macaques and humans, as well as terminal agonal respirations. While the NHPs in our study demonstrated increased respiratory rate and decreased PCO_2_, possibly indicating attempted compensation for metabolic acidosis as is seen in other acute illnesses. There was little change from baseline pH for most subjects, and although Subject 2 showed a decrease in pH and elevated lactate, the increased PCO_2_ suggests both metabolic and respiratory acidoses on PID 7, the latter of which is indicative of respiratory depression in the terminal stage of infection. However, the HCO_3_ levels measured on the iStat cartridges did not decrease as is typically seen in metabolic acidosis, and we lack additional serum chemistries to show the elevated anion gap that would be expected given the terminal hyperlactatemia observed in all macaques. The elevated lactate levels seen in the late stage of infection are indicative of systemic hypoperfusion secondary to circulatory collapse and pronounced shock.

As a well-recognized characteristic of filoviral illness, coagulopathy has been shown to play an integral role in the disease pathogenesis. Here, we found significantly elevated D-dimer levels beginning on PID 5 and continuing through death, indicating DIC begins prior to observed clinical signs, and continues to play an important role throughout the disease course. Additionally, the recorded increase in PT was evident on PID 6. This is likely a result of both consumption of available coagulation factors as well as decrease in synthetic liver function as indicated by sharply increased liver enzymes, decreasing albumin and histology showing severe hepatocellular necrosis. Of note, thrombocytopenia does not appear to play as significant a role in the coagulopathy in MARV-Ang pathogenesis as it does in Ebola virus infection in macaques, a finding echoed in other studies [16,17,18,23,34]. While a decline in platelets was initially noted, platelets increased with all other cell types on PID 7, possibly corresponding to an increase in cytokine/chemokine activity. The overall inflammatory reaction resulting from MARV infection leads to a pro-coagulant state through a number of different mechanisms, including activation of the TF/VIIa coagulation cascade, as well as depletion of protein C and protein S, which have previously been shown to decrease in filovirus infection [18,27,36]. Similarly, septic patients develop DIC through activation of the TF/VIIa pathway via endothelial damage from toxins or cytokine release, as well as concomitant decreases in protein C and protein S, which leads to widespread fibrin deposition and the formation of microthrombi [36].

Central to the pathophysiology of the systemic inflammatory response syndrome and DIC is the hypersecretion of pro-inflammatory cytokines/chemokines. The elevated cytokines seen in this study—G-CSF, IL-1ra, IL-2, IL-1β, IL-5, IL-6, IL-18, MCP-1, MIP-1α, MIP-1β, TNF-α—pointed to a pro-inflammatory cytokine event similar to that previously described for filovirus infection [16,21,22,26,31,37]. In humans, increases in certain cytokine levels have been associated with increased mortality in humans infected with Ebola virus [26,38]. In early sepsis, cytokine hypersecretions are associated with endothelial leakage and vasodilation leading to hypoperfusion, and an aberrant immune response. Our results indicate a similar response to MARV-Ang infection in rhesus macaques, particularly with the terminal increases in TNF-α and IL-6. Interestingly, Subject 1 demonstrated decreases in most of the measured cytokines in the last day of life. Rubins *et al.* reported terminal decreases in cytokine levels during the course of Ebola virus infection in macaques [22], a finding also reported in human MARV infection [31].

Many comparisons have already been made of the presentations of filoviruses and their similarity, both clinically and pathologically, to sepsis [12,14,23,27,31,39,40], and this study supports that viewpoint. Here, we demonstrated that infection with MARV-Ang leads to systemic inflammatory response syndrome marked by fever, tachycardia, and tachypnea, which progresses over several days to include lymphopenia through lymphocyte apoptosis, pro-inflammatory cytokine response including the development of DIC, the onset of hypotension and, finally, multiple organ failure and death. The course of MARV-Ang infection reported in this study parallels what is currently known about the course of human disease (Table 2), and demonstrates that aerosol infection in rhesus macaques is an accurate model of infection for qualification under the FDA “animal rule”. Importantly, by understanding the pathophysiology of MARV-Ang infection through the use of telemetry devices, this potentially opens avenues for further therapeutic research for management of clinical disease after the onset of signs.

## Figures and Tables

**Table 1:** Animal data, challenge dose, peak viremia, and outcome of nonhuman primate subjects exposed to Marburg Angola.

Subject	Sex	Weight (kg)	Challenge Dose (pfu)	Peak Virus Load (RT-PCR) Copies/mL	Peak Viremia (Plaque Assay) pfu/mL	Died/Euthanized (Days Post Infection)	Died/Euthanized (Days via Telemetry)
1	M	7.1	675	2.6 × 10^8^	3.4 × 10^7^	8	7.9
2	F	5.7	7630	5.6 × 10^8^	1.1 × 10^8^	7	7.1
3	M	6.7	1130	1.9 × 10^8^	3.0 × 10^7^	8	7.8
4	F	6.4	1320	3.4 × 10^8^	4.7 × 10^7^	7	7.4
5	M	5.7	1290	5.8 × 10^8^	1.1 × 10^8^	7	7.4

**Table 2:** Summary of Clinical and Laboratory Findings Compared with Presence Documented in Human MARV Disease. Incubation period: exposure to onset of sustained fever (>1.5 °C for 2 h). Early clinical period: onset of sustained fever to onset of sustained tachycardia or tachypnea (>3 SD above baseline for 2 h). Late clinical period: onset of sustained tachycardia/tachypnea to onset of sustained hypotension (>3 SD decrease from baseline for 2 h). Terminal period: onset of sustained hypotension to death or euthanasia.

Macaque Disease (Period)					
Clinical Findings	Incubation	Early Clinical	Late Clinical	Terminal	Human Disease *
Fever	0/5 **	5/5	5/5	5/5	Yes
Tachycardia	0/5	0/5	4/5	5/5	Yes
Tachypnea	0/5	0/5	5/5	5/5	Yes
Hypotension	0/5	0/5	0/5	5/5	Yes
Hypothermia	0/5	0/5	0/5	3/5	Yes
Viremia	2/5	5/5	5/5	5/5	Yes
Lymphopenia	5/5	5/5	5/5	2/5	Yes
↓ Platelets	2/5	2/5	5/5	0/5	Yes
↑ AST, ALT	0/5	0/5	5/5	5/5	Yes AST > ALT
↑ GGT	0/5	0/5	5/5	5/5	Yes
↑Total bilirubin	0/5	0/5	1/5	5/5	Rare
↑ BUN/Cr	0/5	0/5	0/5	2/5	Yes-terminal
↓ Ca^2+^	0/5	0/5	0/5	2/5	Yes-terminal
↓ Albumin	0/5	0/5	0/5	1/5	Not reported
↑ D-dimer	0/5	0/5	4/5	4/5	Yes
Lactic acidosis	0/5	0/5	0/5	1/5	Possible
Fatality				5/5	~90%
Clinical Duration	3.4–4.4 days	2.0–3.0 days		5.5–29.5 h	3–12 day incubation period; median survival = 9 days

* Reference [2,12,14]; ** Number of subjects with clinical finding/total number of subjects.

## Supplementary Files

Supplementary File 1
