# Supplementary material for: Natural History of Aerosol Exposure with Marburg Virus in Rhesus Macaques
Source: Viruses. 2016 Mar 30;8(4):87. doi: 10.3390/v8040087 (PMC4848582; doi:10.3390/v8040087)
Supplement: Supplementary File 1 [file viruses-08-00087-s001.pdf]

Supplementary Materials: Natural History of Aerosol Exposure with Marburg Virus in Rhesus Macaques

Evan C. Ewers, William D. Pratt, Nancy A. Twenhafe, Joshua Shamblin, Ginger Donnelly, Heather Esham, Carly Wlazlowski, Joshua C. Johnson, Miriam Botto, Lisa E. Hensley and Arthur J. Goff

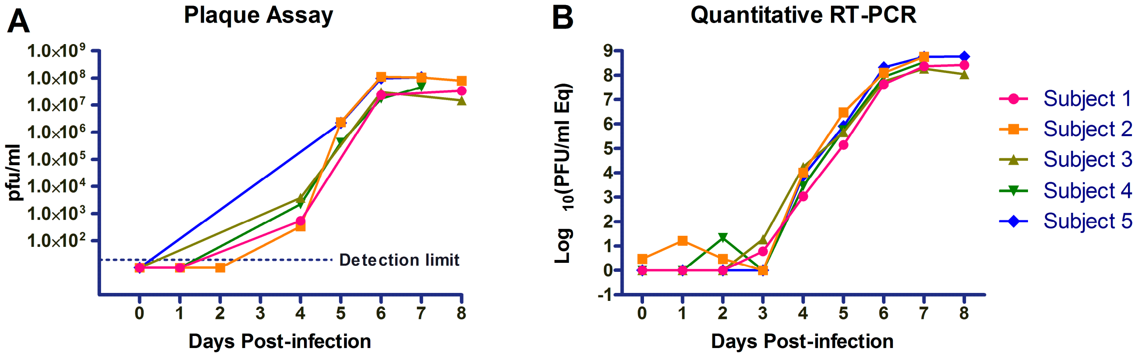

Figure S1. Viremia and virus load as measured by (**A**) plaque assay and (**B**) quantitative RT-PCR.

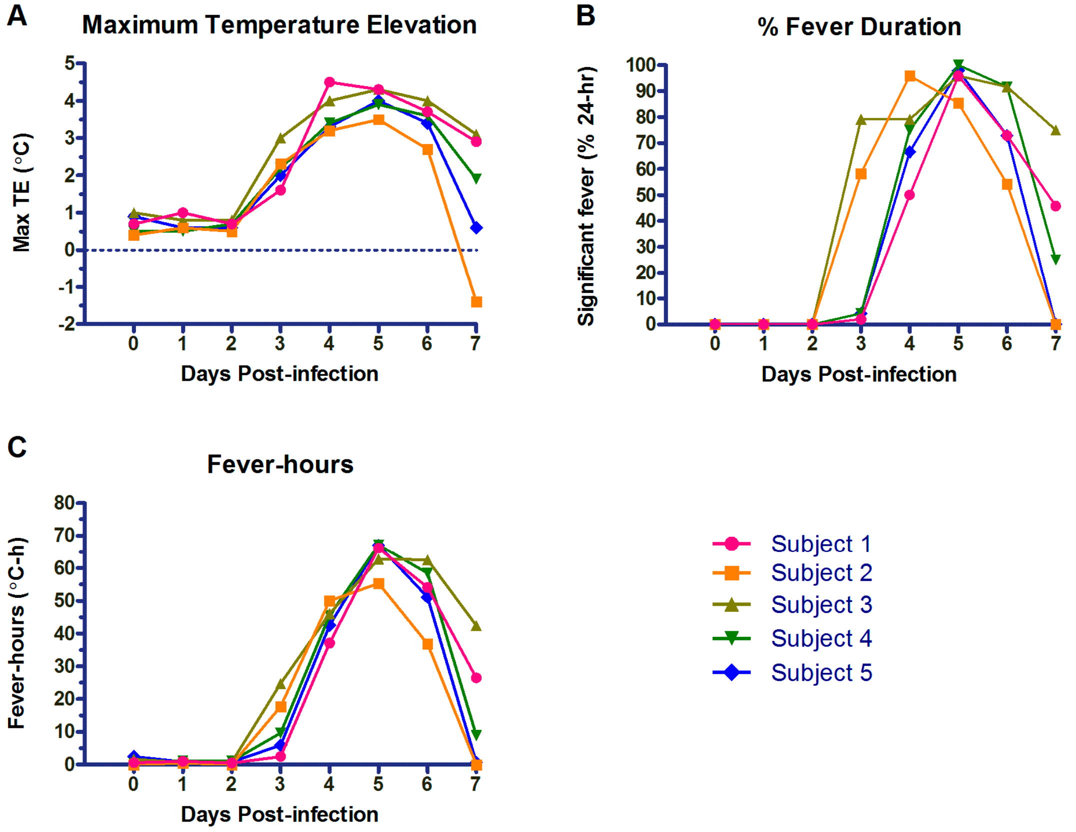

Figure S2. Measurements of fever experienced by macaques following virus challenge. (**A**) Maximum temperature elevation during that 24 h daily time period; (**B**) Percent fever duration, measured as the percent of recorded 24 h daily time period where 30-min interval averages of body temperature were significantly elevated (+3SD) above baseline; (**C**) Fever-hours demonstrating the sum of significant temperature elevation values, and is used to measure the intensity of the fever by approximating the area under the curve.

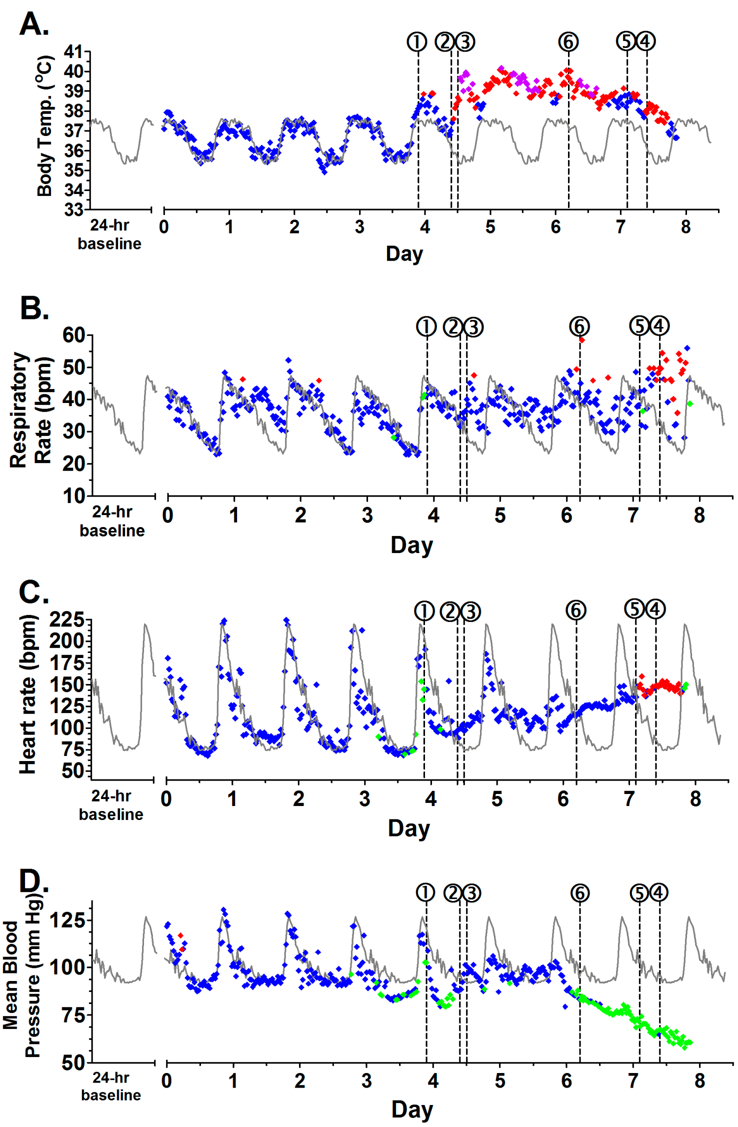

Figure S3. Running plot of body temperature, respiratory rate, heart rate, and blood pressure for Subject 1. (**A**) Body temperature values displaying normal (♦), fever (♦), hyperpyrexia (♦), and hypothermia (♦); (**B**) Respiratory rate; (**C**) Heart rate; (**D**) Blood pressure. Values + 3 SD (♦) or − 3 SD (♦) from baseline were statistically significant; Values < 3 SD (♦) were not significant. Baseline values are seen in gray (–). Circled numbers indicate start of significant response for temperature (1), fever (2), hyperpyrexia (3), respiratory rate (4), heart rate (5), and blood pressure (6).

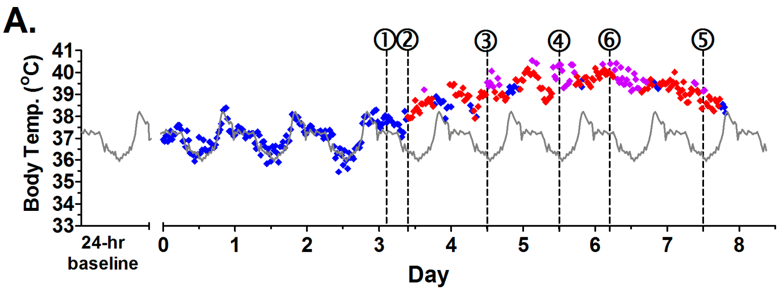

Figure 3. *Cont.*

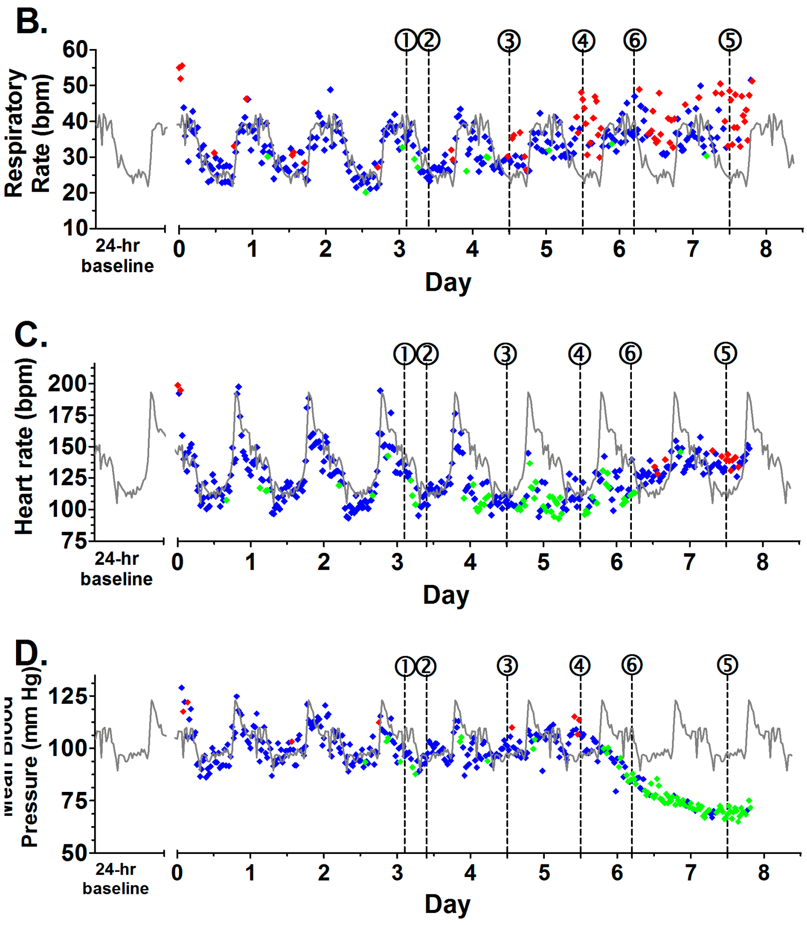

Figure S4. Running plot of body temperature, respiratory rate, heart rate, and blood pressure for Subject 3. (**A**) Body temperature values displaying normal (♦), fever (♦), hyperpyrexia (♦), and hypothermia (♦); (**B**) Respiratory rate; (**C**) Heart rate; (**D**) Blood pressure. Values + 3 SD (♦) or − 3 SD (♦) from baseline were statistically significant; Values < 3 SD (♦) were not significant. Baseline values are seen in gray (–). Circled numbers indicate start of significant response for temperature (1), fever (2), hyperpyrexia (3), respiratory rate (4), heart rate (5), and blood pressure (6).

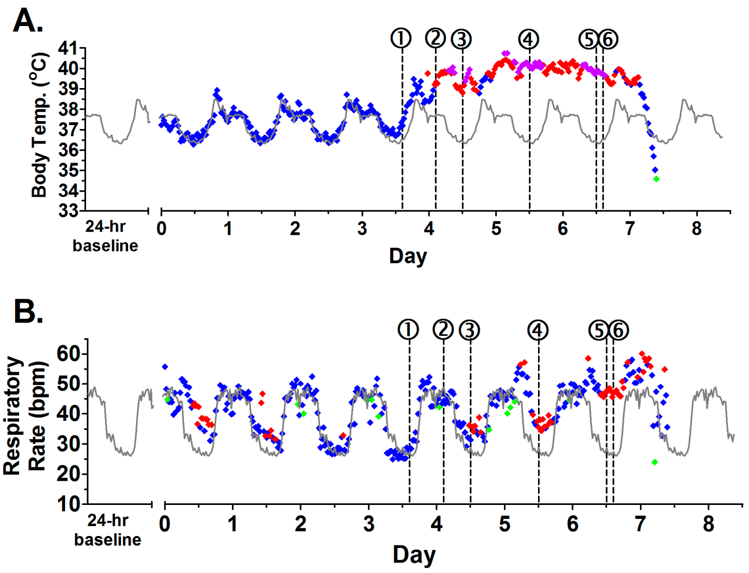

Figure 5. *Cont.*

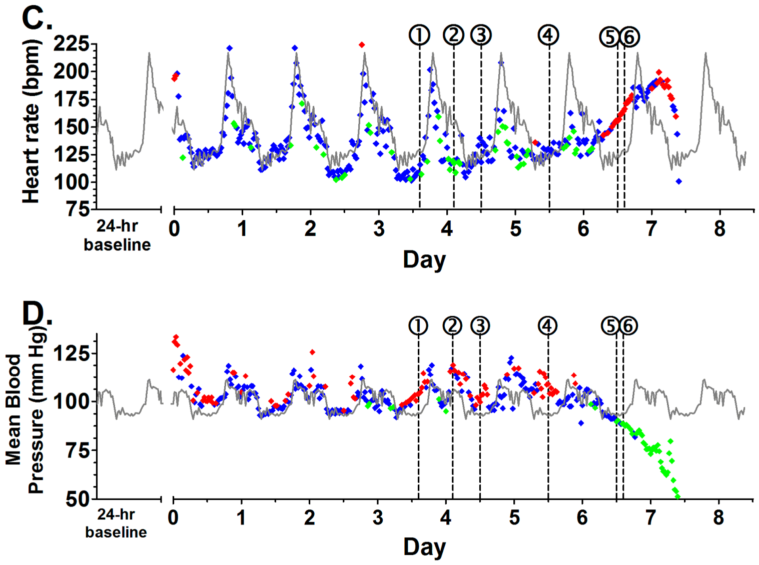

Figure S5. Running plot of body temperature, respiratory rate, heart rate, and blood pressure for Subject 4. (**A**) Body temperature values displaying normal (♦), fever (♦), hyperpyrexia (♦), and hypothermia (♦); (**B**) Respiratory rate; (**C**) Heart rate; (**D**) Blood pressure. Values + 3 SD (♦) or − 3 SD (♦) from baseline were statistically significant; Values < 3 SD (♦) were not significant. Baseline values are seen in gray (–). Circled numbers indicate start of significant response for temperature (1), fever (2), hyperpyrexia (3), respiratory rate (4), heart rate (5), and blood pressure (6).

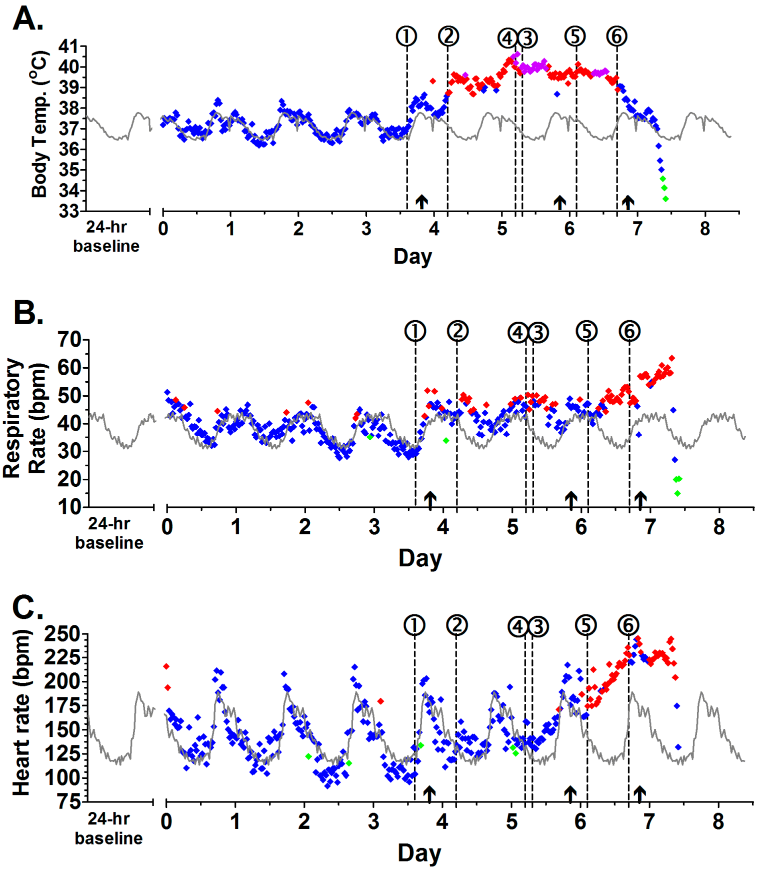

Figure 6. *Cont.*

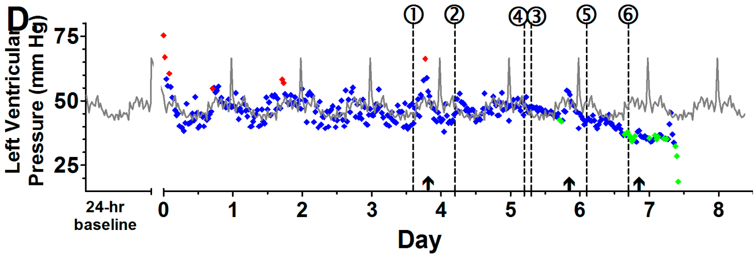

Figure S6. Running plot of body temperature, respiratory rate, heart rate, and blood pressure for Subject 5. (**A**) Body temperature values displaying normal (♦), fever (♦), hyperpyrexia (♦), and hypothermia (♦); (**B**) Respiratory rate; (**C**) Heart rate; (**D**) Blood pressure. Values + 3 SD (♦) or − 3 SD (♦) from baseline were statistically significant; Values < 3 SD (♦) were not significant. Baseline values are seen in gray (–). Circled numbers indicate start of significant response for temperature (1), fever (2), hyperpyrexia (3), respiratory rate (4), heart rate (5), and blood pressure (6). A arrow (↑) marks the time when macaque was anesthetized with ketamine (10 mg/kg) for blood draw.

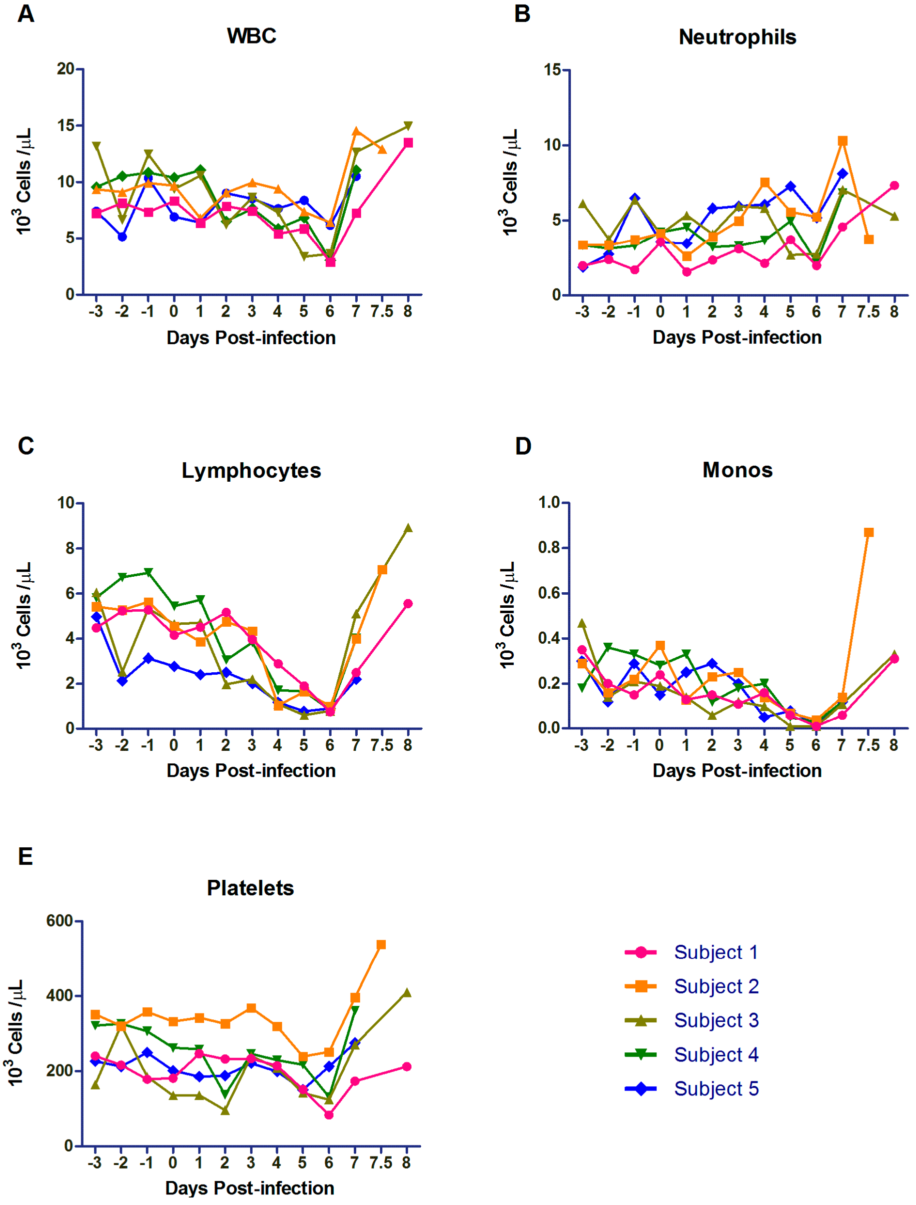

Figure S7. Leukocyte and thrombocyte changes following exposure to MARV-Ang. Graphs show daily trends in (**A**) WBC; (**B**) Neutrophils; (**C**) Lymphocytes; (**D**) Monocytes; and (**E**) Platelets levels in the blood.

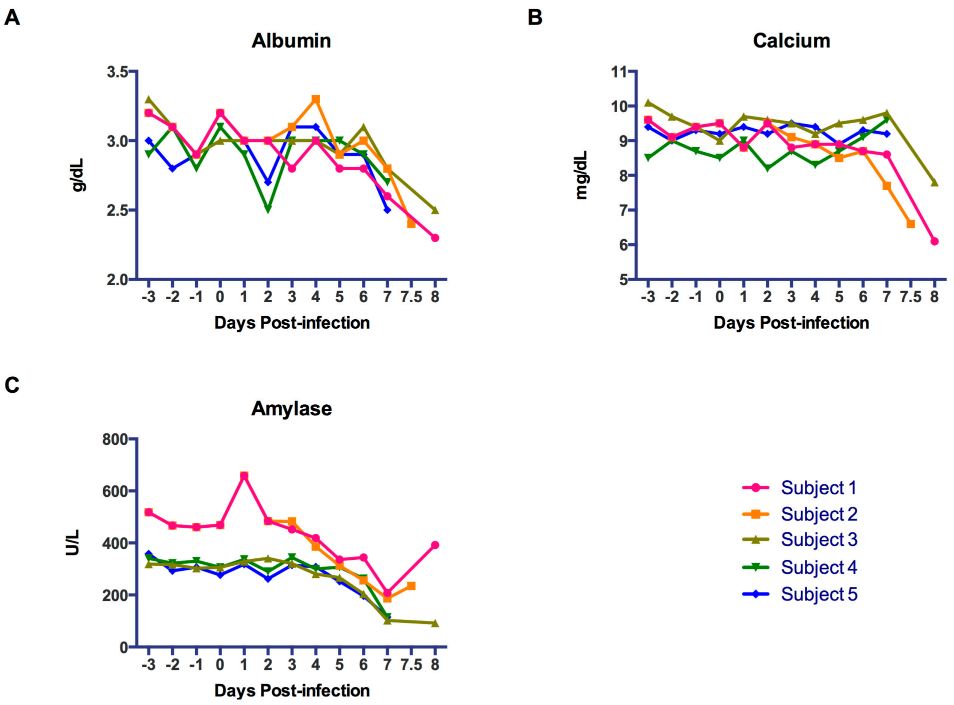

Figure S8. Clinical chemistries: (**A**) Albumin; (**B**) Calcium; and (**C**) Amylase.

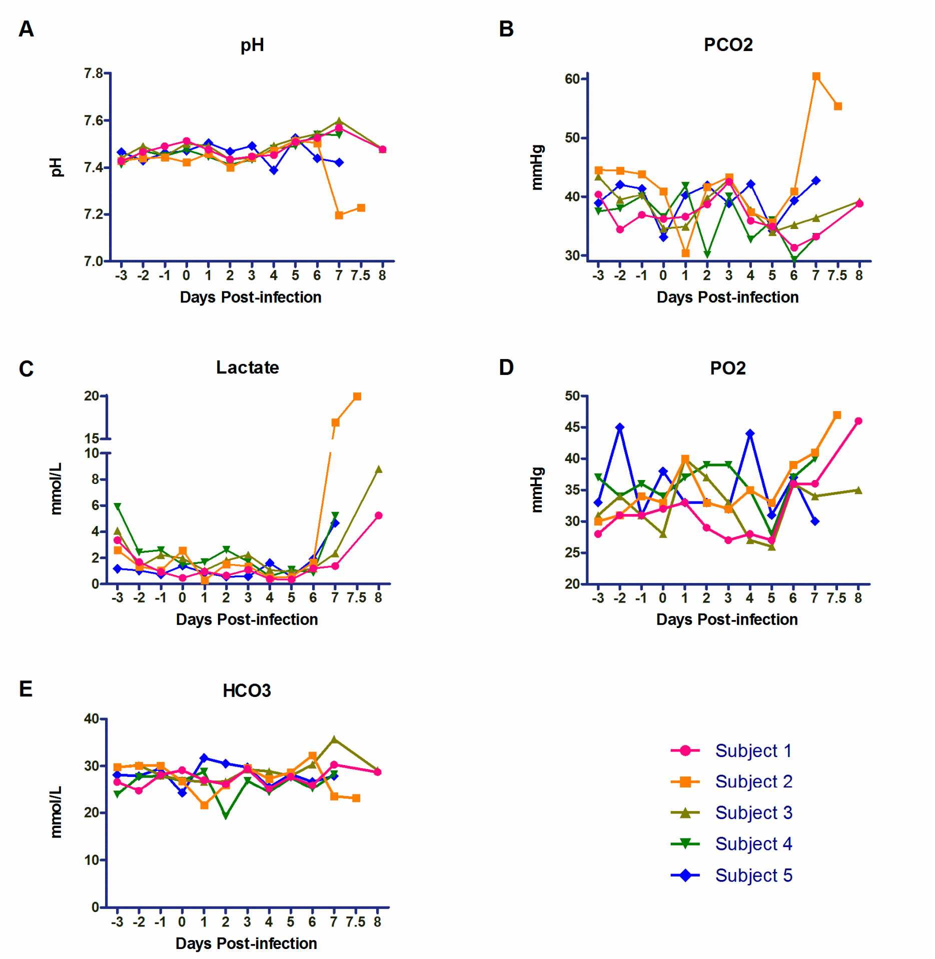

Figure S9. Blood gases measurements. Graphs show daily trends in (**A**) pH; (**B**) PCO_2; (**C**) lactate; (**D**) PO_2, and (**E**) HCO_3 levels in the blood.

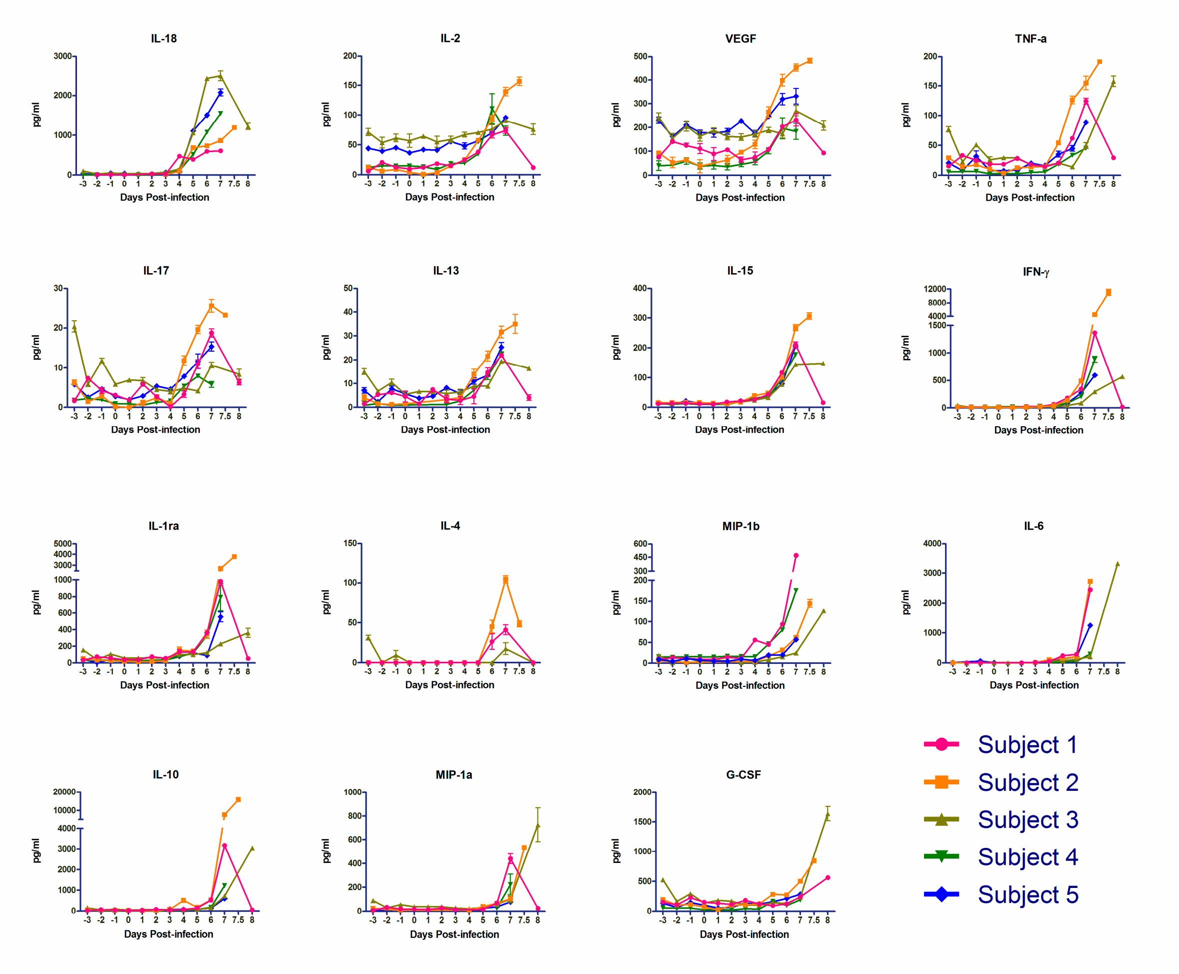

Figure S10. Selected cytokine levels. Graphs show daily trends in IL-18, IL-2, VEGF, TNF-a (top row from left); IL-17, IL-13, IL-15, IFN-γ (second row from left); IL-1ra, IL-4, MIP-1b, IL-6 (third row from left); and IL-10, MIP-1a, G-CSF (bottom row from left) levels in the blood. Samples were run in triplicate, with error bars represent standard deviation.

Table S1. Immunohistochemistry Findings from Rhesus Macaques Challenged by Aerosol with MARV [1].

		Lymph Node						
**Subject**	**Endpoint [2]**	**Tracheo-Bronchial**	**Mediastinal**	**Inguinal**	**Spleen**	**Liver**	**Lung**	**Skin**
1	E	+++	++	++	+++	+++	++	-
2	E	+++	++	+	+++	+++	+	-
3	D	++	++	+	+++	+++	+	-
4	D	+++	++	+	+++	+++	+	-
5	E	+++	++	+	+++	+++	+	-

[1] Changes not present, + = minimal to mild changes, ++ = moderate changes, +++ = marked changes; [2] Endpoint: died (D) or euthanized (E).

© 2016 by the authors; licensee MDPI, Basel, Switzerland. This article is an open access article distributed under the terms and conditions of the Creative Commons by Attribution (CC-BY) license (http://creativecommons.org/licenses/by/4.0/).
